# The apple does not fall far: stable predictive relationships between parents’ ratings of their own and their children’s self-regulatory abilities

**DOI:** 10.1186/s13034-024-00814-z

**Published:** 2024-10-03

**Authors:** Johanna Kneidinger, José C. García Alanis, Ricarda Steinmayr, Silvia Schneider, Hanna Christiansen

**Affiliations:** 1 Department of Psychology, Philipps-University Marburg, Marburg, Germany; 2 Department of Psychology, TU Dortmund, Dortmund, Germany; 3 Department of Psychology, Ruhr University Bochum, Bochum, Germany

**Keywords:** Executive function, Delay aversion, Parental influence, Child development

## Abstract

Having control over your own behavior and impulses is a critical skill that influences children’s academic, social, and emotional development. This study investigates the stability and predictive relationships between parents’ ratings of their own and their children’s executive function and delay aversion. Using data from approximately 1700 families collected during the COVID-19 pandemic, we employed hierarchical structural equation models and cross-lagged panel models to analyze the temporal stability and directional influences of executive function and delay aversion assessments.

Our analysis revealed a substantial latent correlation (*r* = 0.48, *p* < 0.001) between parents’ and children’s executive function problems, indicating a shared variance of approximately 23%. Significant cross-lagged effects were found, with parental executive functions at T1 predicting child executive functions at T2 (β = 0.16, *p* = 0.005). For delay aversion, we found a latent correlation of *r* = 0.53 (*p* < 0.001) and significant within-timepoint and temporal stability, but no cross-lagged effects.

These findings suggest that higher levels of executive function problems reported by parents at T1 correspond to an increased perception of similar problems in their children at T2. This highlights the importance of parental self-perception in assessing children’s abilities. Our results highlight the importance of incorporating family dynamics into interventions targeting executive function difficulties and delay aversion in children, and understanding this interplay enables the development of more effective, individualized approaches to support positive developmental outcomes.

## Background

Self-regulatory abilities crucially impact human development throughout all of development [1]. These abilities refer to the process of willingly controlling and adapting one’s actions to achieve short- and long-term goals [2]. Beyond that, self-regulatory abilities are a fundamental aspect of human functioning, involving the dynamic process of setting a desired goal, taking actionable steps toward it, and continuously monitoring progress. This concept can be clearly observed in real-life situations [2]. For example, in a classroom setting, a child who manages to focus on completing a challenging math problem while resisting the urge to move on to more appealing activities is demonstrating self-regulation. This ability involves various subprocesses, including the intentional control and coordination of thoughts and behaviors, as well as managing physiological responses to maintain calmness and focus under potential stress [3]. Self-regulation is multifaceted and spans multiple domains of human behavior and experience [2, 3] including *Executive Function* and *Delay Aversion* that both capture complementary facets of self-regulation [4] and are in focus of empirical research [5]. Executive Function emphasizes the dynamic cognitive mechanisms that facilitate humans’ capacity to focus attention on relevant characteristics of an ongoing task and inhibit distractions [6, 7]. Conversely, Delay Aversion refers to individuals’ inclination towards favoring immediate rewards over delayed ones, presumably to avoid the aversive sensation of waiting [8]. Specifically, it has been proposed that reduced inhibitory control (part of the pathway of executive dysfunction) and increased Delay Aversion (where boring waiting situations are avoided if possible or escaped through impulsive behavior) negatively affect self-regulatory abilities [9]. Indeed, empirical data indicate that clinically relevant impairments in self-regulated action, such as those observed in children diagnosed with attention-deficit/hyperactivity disorder (ADHD), are substantiated by a maladaptive interplay of deficient Executive Functions and the emergence of Delay Aversion [10–12]. Further, children who are better at controlling attention, inhibiting behavior, and regulating emotions tend to show fewer conduct problems [13] and achieve better psychosocial, and mental health outcomes later in life [1, 14]. Moreover, children’s executive functions play a main role in the context of short- and long-term academic outcomes, such as early mathematics, language, and literacy skills [15]. However, many traditional approaches for assessing those skills in children rely on caregiver ratings [16, 17], which have been shown to be susceptible to interpersonal dynamics between caregivers and children [18]. In addition, concerns regarding the temporal stability and predictive validity of these measures [16] remain unaddressed. Understanding the structural covariance and temporal stability of caregiver assessments is therefore essential for developing better interventions and fostering executive functions and delay aversion in more individualized settings.

### Caregivers’ influence on children’s regulatory capacity

The ability of willingly controlling and adapting one’s actions experiences rapid growth during early childhood [19] and is essential for a successful transition to formal schooling [20]. In a school setting, teachers play a pivotal role in fostering children’s executive functions as well as their ability to withstand delayed reward. They not only introduce children to self-regulated learning, but also provide valuable reinforcers and instructions through their own regulatory skills [21], aiding students in task mastery and goal achievement.

In recent years, however, the COVID-19 pandemic showed how disruptions in daily routines and transitioning learning environments to more home-based settings can pose significant challenges for children and adolescents [22, 23]. Many students encountered difficulties in structuring their day, initiating learning sessions, and maintaining focus on their assignments [24], partly due to the nature of distance learning characterized by reduced teacher support. This led to an increased risk of students missing out on broader learning opportunities and feeling overwhelmed by academic demands [25]. Children who engaged in distance learning were more reliant on their parents to initiate and maintain self-regulated academic activities [26, 27]. Additionally, those who found distance learning more challenging were less likely to work independently and often required additional assistance from caregivers to cope with academic requirements [23].

Amidst the shift to distance learning during the pandemic, many parents assumed a crucial role in fostering self-regulatory abilities, effectively stepping in as surrogates for teachers [28]. Many families had to manage the added responsibility of helping their children maintain academic focus, structure their routines, and sustain their motivation within the learning process [28]. Such collaboration can be viewed as a co-regulation process [21, 29], heavily reliant on the self-regulatory process of the co-regulators, in this case, parents and their children [29]. Co-regulation, where parents or teachers guide children’s thoughts, behaviors, or emotions to align with certain expectations, is crucial in helping children gradually internalize these strategies [30]. As children grow older, co-regulation processes expose them to increasingly complex experiences, allowing them to practice self-regulation within relational contexts and develop behavior patterns that solidify into regulatory abilities over time [31]. The process of internalization is considered the key mechanism for transforming co-regulation into self-regulation [32].

Accordingly, family environment plays a pivotal role in the development of children’s executive functions, with parental characteristics influencing both the family environment and children’s development [15]. While hereditary factors partially explain differences in children’s executive functions, several other factors, including shared genetic influence, biological influences, and environmental as well as social factors, also contribute to the link between parental and children’s executive functions [23, 28]. Additionally, key mechanisms such as parental autonomy support and sensitive caregiving practices have been identified as critical for the transmission of executive function from parent to child [33, 34]. Given the importance of these environmental and social factors, this raises the question of whether there are other, more nuanced elements, such as parental beliefs, that might also play a significant role. Additionally, Murphey [35] states in his model that parental beliefs can affect how parents perceive their children’s characteristics as well as moderate their responses accordingly.

### The present study

Despite emerging research emphasizing the importance of parental self-regulatory capacity during child development [36] and its potential impact on children’s academic, social, motivational, and emotional trajectories [37], the relationship between parent-child self-regulatory abilities is not well understood [38–40]. Although it has been shown that there is a connection between parental and child self-regulatory skills [41–43] and that parental attributions and expectations influence their children’s treatment progress [44], some questions require further investigation. In particular, the relationship between how parents view their own self-regulation skills and their perceptions of their child’s self-regulation requires further elucidation [45]. A better understanding of these relationships can provide insight into the reinforcement and coupling mechanisms that shape the development of cognitive abilities in children and adolescents [38]. Such insights are crucial for helping researchers and practitioners design better and more individualized interventions that promote positive developmental outcomes [29].

To address these questions, we sought to estimate the latent correlation between parents’ assessments of their own and their children’s executive functions and delay aversion. Furthermore, we examined the temporal stability of these ratings, assessing whether initial ratings predicted parent assessments later in time. Specifically, we tested how parents’ initial ratings of their own deficits predicted their subsequent assessments of both their own deficits and those of their children. Conversely, we also tested whether initial ratings of their children’s deficits predicted later assessments of their children’s deficits and the parents’ own deficits. To accomplish this, we analyzed a large dataset of parental self-report assessments of their own and their children’s difficulties in lower-level domains of cognitive-emotional regulation.

The self-report data analyzed in the present study were collected from approximately 1700 families during the COVID-19 pandemic. The data spanned two measurement time points separated by several months. We used hierarchical structural equation models (SEM) to estimate the latent correlation between parents’ and their children’s executive functions and delay aversion across multiple measurements. Additionally, we employed a cross-lagged panel model to assess the directional influence of parents’ initial ratings of executive functions and delay aversion on their later scores. This analysis aimed to determine whether parental assessments of self-regulatory ability significantly predicted later assessments and whether these influences were specific to the assessment target (either their own or their children’s abilities) or generalized across targets (i.e., establishing cross-lagged relationships). Through these analyses, we aimed to contribute valuable insights into the interplay of parental and child executive functions and delay aversion as well as their potential long-term effects.

## Methods

### Procedure

Data were collected from seven European countries through an anonymous digital survey [46]. The survey aimed to understand parental experiences with distance learning and prompted parents to assess both their own and their children’s executive functions and delay aversion during the pandemic [27, 47, 48]. In the present manuscript, we exclusively analyzed the German data subset, as Germany was the only country where data was collected across two measurement time points. Therefore, data collection occurred in two phases. The initial survey phase spanned from April 28th to November 1st, 2020 (assessment timepoint one - T1), followed by a second phase from December 6th, 2020, to February 25th, 2021 (assessment timepoint two - T2). The survey was distributed to parents through various channels. During T1, it was promoted via social media, school blackboards, parent networks, and support groups. For T2, parents received invitations via email. The data needed to reproduce the analysis and results reported here can be accessed through the supplementary materials repository provided on the open science framework (https://osf.io/rc934/?view_only=5cb3c5e1d5aa4dc0bf25aa78c752dc3c).

### Participants

To be eligible for participation, respondents had to be parents of children or adolescents aged between five and 18 years, enrolled in standard schooling, and transitioning to distance learning due to pandemic-induced school closures. Initially, 1,767 parents participated at T1, and 1,082 at T2. After excluding mismatched data, entries with errors, and parents with children aged older than 18 years, the analyses were based on data from 1,655 participants at T1 and 537 participants at T2.

**Table 1:** Sample descriptives for both measurement timepoints

	Measurement timepoint	
	T1	T2
Families N	1655	537
Parents		
N males	231	68
N females	1421	468
N others*	3	1
Mean age (SD)	43.04 (6.10)	43.73 (6.18)
Age range	23–68	28–68
Children		
N males	860	281
N females	793	256
N others*	2	-
Mean age (SD)	11.45 (3.01)	12.01 (3.04)
Age range	5–17	5–18

*SD* Standard deviation, *N*  Number of cases

*Others contains diverse, intersexual and unassignable

Descriptive statistics are provided in Table 1. The average age of the children was 11.45 years at T1 and increased to 12.01 years by T2. Female children represented 47.92% of the sample at T1 and 47.67% at T2. The mean age of parents at T1 was 43.04 years, with females comprising 85.86% of the sample. By T2, the average parental age was slightly higher at 43.73 years, with females comprising 87.15% of the participants.

### Instruments

The online survey assessed various facets of parental experiences during distance learning. Additionally, parents were asked to rate their own and their children’s self-regulatory skills by indicating their agreement or disagreement (1 = “strongly disagree” to 5 = “strongly agree”) with a series of statements about their own and their children’s daily difficulties with executive functions and control over delay aversion. A higher score indicated more pronounced executive function problems and increased delay aversion. The tools utilized for these measurements are elaborated upon in the subsequent sections.

#### Childhood Executive Functioning Inventory (CHEXI)

To assess children’s executive function problems, the survey included an abbreviated version of the Childhood Executive Functioning Inventory - CHEXI [49]. The CHEXI, freely available in many languages (www.chexi.se), includes two subscales measuring difficulties in working memory (e.g., “when asked to do several things, he/she only remembers the first or last”) and the inhibition domain (e.g., “has difficulty holding back his/her activity despite being told to do so”). The online survey comprised eight items: four for working memory and four for inhibition. Working memory items showed good internal consistency at T1 (Cronbach’s alpha = 0.86, 95% CI = [0.85–0.87]) and T2 (Cronbach’s alpha = 0.88, 95% CI = [0.87–0.89]). The same was the case for inhibition items at T1 (Cronbach’s alpha = 0.84, 95% CI = [0.83–0.85]) and T2 (Cronbach’s alpha = 0.85, 95% CI = [0.83–0.86]). Although specific psychometric data for the German version of this tool are not available, evidence from the Persian adaptation suggests that the CHEXI exhibits robust psychometric properties across cultures. The Persian version confirmed a two-factor structure (working memory and inhibition) and demonstrated high internal consistency, test-retest reliability, and measurement invariance by sex and age. Additionally, the study provided evidence of adequate convergent and known-group validity.

#### Adult Executive Functioning Inventory (ADEXI)

To measure parental executive function problems, the survey included an abbreviated version of the Adult Executive Functioning Inventory– ADEXI [50]. Like the CHEXI, the ADEXI is freely available in various languages (www.chexi.se) and includes two subscales measuring difficulties in working memory (e.g., “when someone asks me to do several things, I sometimes remember only the first or last”) and the inhibition domain (e.g., “I have a tendency to do things without first thinking about what could happen”). The online survey comprised eight items: four for working memory and four for inhibition. Working memory items showed acceptable internal consistency at T1 (Cronbach’s alpha = 0.79, 95% CI = [0.77–0.81]) and T2 (Cronbach’s alpha = 0.77, 95% CI = [0.76–0.79]). In contrast, the internal consistency of inhibition items was somewhat lower at T1 (Cronbach’s alpha = 0.61, 95% CI = [0.57–0.64]) and T2 (Cronbach’s alpha = 0.62, 95% CI = [0.60–0.65]). No specific psychometric data are available for the German version of the ADEXI. However, the Spanish adaptation demonstrates robust psychometric properties across cultures. Confirmatory Factor Analysis confirmed a two-factor structure (working memory and inhibition) and revealed high internal consistency (α = 0.87) and significant correlations with relevant measures, supporting its construct validity. These results affirm the Spanish version of the ADEXI as a reliable and valid tool for assessing executive functions in non-clinical populations, applicable for both clinical and research purposes.

#### Quick Delay Questionnaire (QDQ)

Child and parental delay aversion were measured using a brief (two-item) version of the Quick Delay Questionnaire – QDQ [51]. Children’s delay aversion items showed acceptable internal consistency at T1 (Cronbach’s alpha = 0.77, 95% CI = [0.74–0.79]) and T2 (Cronbach’s alpha = 0.77, 95% CI = [0.75–0.79]). Similarly, the parents’ delay aversion items showed acceptable internal consistency at T1 (Cronbach’s alpha = 0.76, 95% CI = [0.73–0.78]) and T2 (Cronbach’s alpha = 0.76, 95% CI = [0.73–0.78]).

### Statistical analyses

Before analyses, all variables were converted to z-scores, ensuring each variable had a mean of zero and a standard deviation of one. This was done to mitigate potential effects caused by discrepancies in scale between variables and to avoid potential estimation problems resulting from differing variances between the response variables. The standardized data formed the basis for all subsequent analyses.

We used structural equation models to estimate the latent correlation and longitudinal associations between parental and child executive function deficits, as well as parental and child delay aversion. All models were estimated in the R programming environment, version 4.3.2 [52], using the *lavaan* package [53]. We used the maximum likelihood algorithm with robust Huber-White standard errors and a scaled test statistic (asymptotically) equal to the Yuan-Bentler test statistic to account for possible deviations from multivariate normality. As the variables were standardized, we fixed all estimated indicator means to zero, a fact that informs the degrees of freedom for all reported models. In some specific cases (reported below), the algorithm estimated non-significant residual variances with values below zero. To account for this issue, we refitted the corresponding model with that residual variance fixed to zero. For handling missing data, we used the full information maximum likelihood (FIML) estimator [54, 55] as implemented in the *lavaan* package.

We evaluated goodness-of-fit based on the comparative fit index, CFI [56], and the root mean square error of approximation, RMSEA [57]. We considered CFI values > 0.95 and RMSEA values < 0.06 to indicate good model fit, and CFI values > 0.90 and RMSEA values < 0.08 to indicate acceptable model fit, as recommended by Brown and Cudeck [57] and Hu and Bentler [58]. Effects were considered statistically significant if the p-value was less than α = 0.05.

In a first step, we estimated the measurement and structure models for parents and children at each measurement timepoint separately. The CFA results across the four models consistently show that both working memory and inhibition factors significantly contribute to the higher-order executive function construct. Working memory loadings ranged from 0.554 to 0.862, with confidence intervals (CIs) from 0.435 to 0.895, while inhibition loadings ranged from 0.352 to 0.862, with CIs from 0.272 to 0.915. The strongest contributions were observed in the models based on child ratings. Regarding delay aversion, the CFA results showed strong and statistically significant loadings for all items, with loadings ranging from 0.779 to 0.793 and confidence intervals indicating a robust relationship between the observed variables and the latent factors. For delay aversion, the models fit well for the overall sample. However, there were notable fit issues when the model was fitted to specific subgroups, particularly younger children (< 11 years old), where CFI and RMSEA suggest the model may not fully capture the underlying processes. These findings suggest that while the delay aversion constructs are generally well-represented, refinements may be necessary to account for developmental differences across age groups. We provide the complete solution for each model (overall sample and age subgroups) on the OSF. Please refer to the supplemental results for further inspection of these models (https://osf.io/rc934/?view_only=5cb3c5e1d5aa4dc0bf25aa78c752dc3c).

Based on these results, we estimated four different models. In Model 1 and Model 2, we assumed a hierarchical structure for executive function problems: Parents’ and their children’s executive functions were modeled as measurement timepoint-specific and parent and child-specific higher-order factors. The working memory and inhibition sub-facets of the CHEXI and ADEXI were estimated to conform to the measurement timepoint-specific and parent and child-specific lower part of the factor hierarchy (i.e., subdomains). The higher-order factors executive function (problems) at timepoint 1 (EF T1 parent and EF T1 child) and executive function (problems) at timepoint 2 (EF T2 parent and EF T2 child) can be interpreted as the common variance shared by the working memory and inhibition subdomains at each measurement timepoint, which are thought to be correlated. In Model 1, we estimated a general trait factor for parental and child executive function problems that integrated the measurement timepoint-specific executive function problems factors. This is equivalent to the assumption that the common variance in executive function problems ratings for parents and their children can each be explained by a single, corresponding trait factor contributing to each of the measurements. Finally, Model 1 estimated the latent correlations between the general trait factor for parental executive function problems and the general trait factor for child executive function problems.

In Model 2, we did not include the general trait factors for parental and child executive function. Instead, we estimated the cross-lagged relationships between the higher-order factors EF T1 parent, EF T1 child, EF T2 parent, and EF T2 child to estimate the directionality and longitudinal associations between parental and child executive functions across measurements. The primary goal of this model was to extend Model 1 and to provide a more fine-grained understanding of the interrelationships between parental and child executive functions both within and between the two measurement timepoints. This model captures correlations within a single measurement timepoint (assessing the initial overlap between parent and child executive function), associations between the same traits measured at different times (allowing us to assess their temporal stability), and relationships between different domains captured at disparate times (allowing us to examine variance in one group of subjects as it may predict changes in the other) (cf. 59).

Model 3 and Model 4 were homologous to Model 1 and Model 2, respectively, but concerned parents’ ratings of their own and their children’s delay aversion. One further difference between the executive function models (i.e., Models 1 and 2) and the delay aversion models (i.e., Models 3 and 4) was that the delay aversion models did not include subdomain factors, as delay aversion was assessed using items from only one scale.

Finally, in supplemental analyses, we tested whether models 1, 2, 3, and 4 showed comparable model fit when computed on age subgroups within the sample (younger vs. older children). All models demonstrated a comparable fit, with minimal differences, indicating a robust relationship between the latent factors across different age groups. Please refer to the supplemental results on the OSF for further inspection of these models. Furthermore, cross-lagged relationships in Models 2 and 4 (i.e., the cross-lagged panel models for executive functions and delay aversion) were moderated by the amount of time parents and their children spent working together on school assignments from home between measurement timepoints. These additional analyses aimed to explore potential moderating effects of the distance learning context on the longitudinal associations between parental and child executive functions and delay aversion. The models showed no substantial moderation effects and can be found in the supplemental analysis section provided on the OSF.

## Results

### Relationship between parental and child executive function

#### Latent correlation

**Fig. 1:**
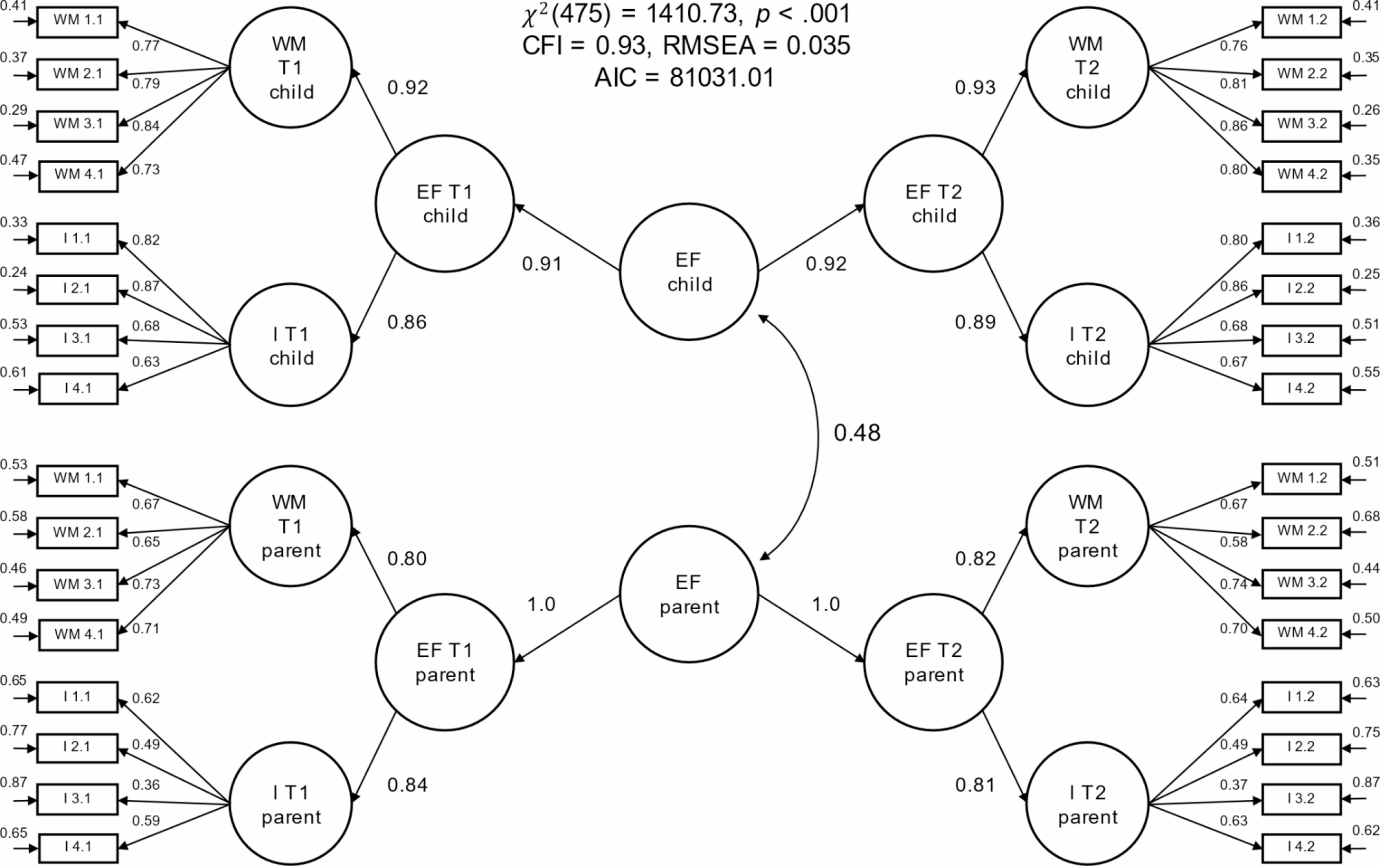
Hierarchical latent correlation model of executive function problems (Model 1). The item initials in the item labels (WM or I) denote whether the item belonged to the working memory or inhibition sub-facet of the executive function questionnaire. The first number in the item labels denotes the item index (1 to 4) and the second number denotes the measurement timepoint (1 or 2). The figure depicts standardized path coefficients and unstandardized residual variances. Residual covariances between the same item measured at different timepoints are omitted from the plot to avoid clutter

Model 1 estimated the latent correlation between two higher-order factors that captured the trait components of parents’ ratings of their own and their children’s executive function problems across measurements (see Fig. 1). Model 1 showed relatively good model fit (χ² [df = 475] = 1410.73, CFI = 0.93, RMSEA = 0.035). The model estimated a substantial latent correlation between the trait factors for parental and child executive function problems of *r* = 0.48 (95% CI = [0.41, 0.55], *p* < 0.001), indicating that parents’ assessment of their own and their children’s executive function problems shared approximately 23% of their variance.

Results indicated that the hierarchical structure in Model 1 captured large proportions of the variance present in the executive function subdomains of working memory (Children: β_T1_ = 0.92, β_T2_ = 0.93; parents: β_T1_ = 0.80, β_T2_ = 0.82) and inhibition (Children: β_T1_ = 0.86, β_T2_ = 0.89; parents: β_T1_ = 0.84, β_T2_ = 0.81), with the proportions of variance explained by the timepoint-specific hierarchical structure being overall somewhat higher for child executive function problems than for parental executive function problems.

#### Cross-lagged relationships

**Fig. 2:**
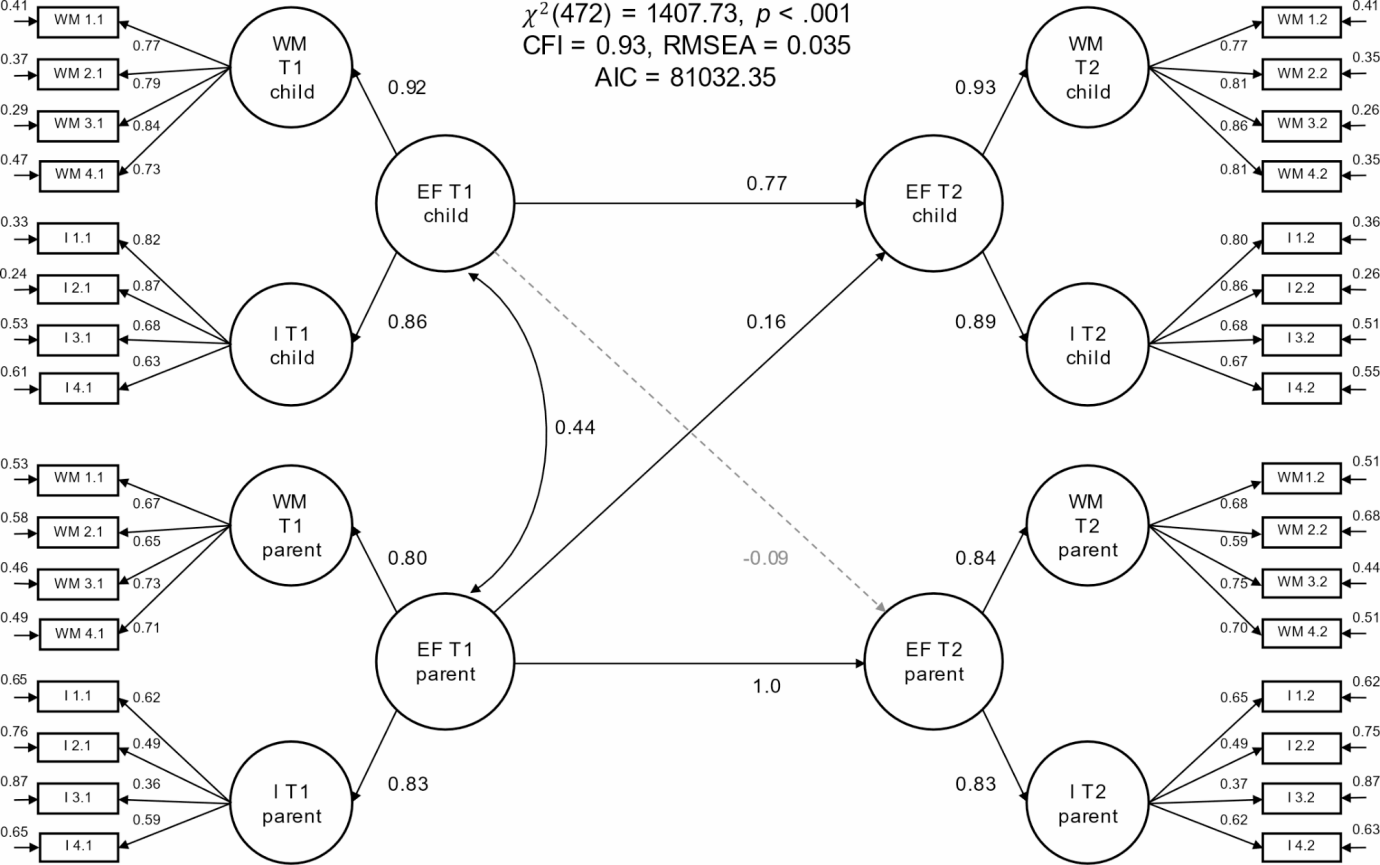
Cross-lagged panel model of executive function problems (Model 2). The item initials in the item labels (WM or I) denote whether the item belonged to the working memory or inhibition sub-facet of the executive function questionnaire. The first number in the item labels denotes the item index (1 to 4) and the second number denotes the measurement timepoint (1 or 2). The figure depicts standardized path coefficients and unstandardized residual variances. We fixed T2 EF residual variance at zero. Residual covariances between the same item measured at different timepoints are omitted from the plot to avoid clutter

 Model 2 estimated the latent relationships between parental and child executive function problems both within and between the two measurement timepoints (see Fig. 2). The model estimated the latent correlation coefficient linking the two higher-order factors that captured the common variance shared by the working memory and inhibition subdomains ratings for child and parent executive function problems at T1. Furthermore, the model estimated the latent regression coefficient between the higher-order factors for child executive function at T1 and T2, parent executive function at T1 and T2, as well as the cross-lagged latent regression coefficients linking child executive function at T1 and parent executive function at T2, and parent executive function at T1 and child executive function at T2. Parental executive function problems at T2 (EF parent T2) were fully accounted for by the model, and thus its residual variance was fixed to zero. The model showed relatively good model fit (χ² [df = 472] = 1407.73, CFI = 0.93, RMSEA = 0.035).

The model estimated a substantial latent correlation between parent and child executive function problems at T1 of *r* = 0.44 (95% CI = [0.38, 0.51], *p* < 0.001), replicating the latent correlation estimated by Model 1. Furthermore, child executive functioning problems at T1 largely predicted child executive functioning problems at T2 (β = 0.77, 95% CI = [0.68, 0.86], *p* < 0.001), but did not predict parental executive functioning problems at T2 (β = -0.09, 95% CI = [-0.22, 0.05], *p* = 0.198). In contrast, parent executive function problems at T1 were highly predictive of parental executive function problems at T2 (β = 1.0, 95% CI = [0.98, 1.00], *p* < 0.001) and were also predictive of child executive function problems at T2 (β = 0.16, 95% CI = [0.05, 0.28], *p* = 0.005).

### Relationship between parental and child delay aversion

#### Latent correlation

**Fig. 3:**
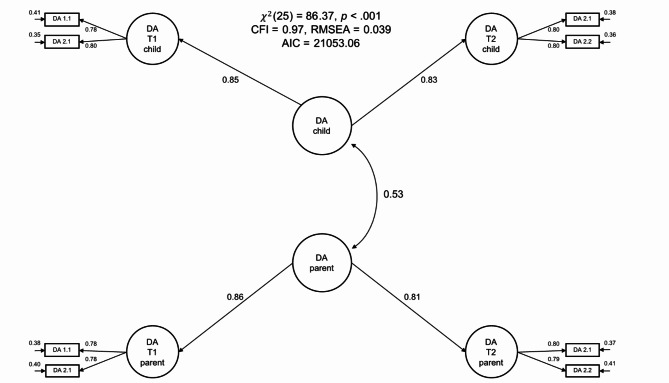
Hierarchical latent correlation model of delay aversion (Model 3). The item initials in the item labels (DA) denote that the items belonged to the delay aversion questionnaire. The first number in the item labels denotes the item index (1 to 2) and the second number denotes the measurement timepoint (1 or 2). The figure depicts standardized path coefficients and unstandardized residual variances. Residual covariances between the same item measured at different timepoints are omitted from the plot to avoid clutter

Model 3 estimated the latent correlation between two higher-order factors that captured the trait components of parents’ ratings of their own and their children’s delay aversion across measurements (see Fig. 3). Model 3 showed good model fit (χ² [df = 25] = 86.37, CFI = 0.97, RMSEA = 0.039). The model estimated a substantial latent correlation between the trait factors of parental and child delay aversion of *r* = 0.53 (95% CI = [0.43, 0.62], *p* < 0.001), indicating that parents’ assessment of their own and their children’s delay aversion shared approximately 28% of their variance.

#### Cross-lagged relationships

**Fig. 4:**
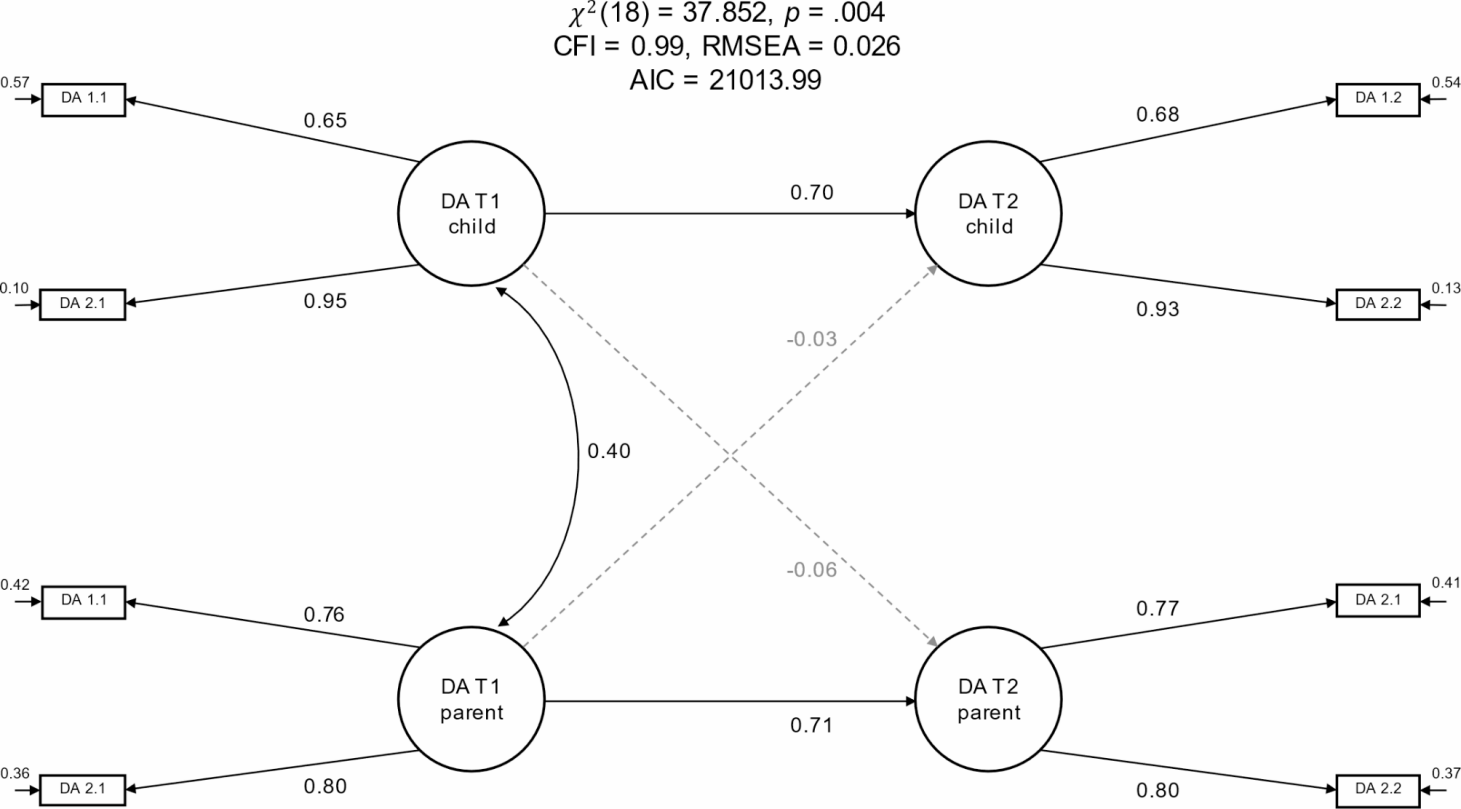
Cross-lagged panel model of delay aversion (Model 4). The item initials in the item labels (DA) denote that the items belonged to the delay aversion questionnaire. The first number in the item labels denotes the item index (1 to 2) and the second number denotes the measurement timepoint (1 or 2). The figure depicts standardized path coefficients and unstandardized residual variances. Residual covariances between the same item measured at different timepoints are omitted from the plot to avoid clutter

Model 4 estimated the latent relationships between parental and child delay aversion both within and between the two measurement timepoints (see Fig. 4). The model estimated the latent correlation coefficient linking the two factors that captured the common variance of the ratings for child and parent delay aversion at T1. Furthermore, the model estimated the latent regression coefficients between the factors for child delay aversion at T1 and T2, parent delay aversion at T1 and T2, as well as the cross-lagged latent regression coefficients linking child delay aversion at T1 and parent delay aversion at T2, and parent delay aversion at T1 and child delay aversion at T2. The model showed good fit (χ² [df = 18] = 37.852, CFI = 0.99, RMSEA = 0.026).

Model 4 estimated a substantial latent correlation between parent and child delay aversion at T1 of *r* = 0.40 (95% CI = [0.33, 0.47]). This estimate was somewhat lower than the overall latent correlation estimated by Model 3. Furthermore, child delay aversion at T1 largely predicted child delay aversion at T2 (β = 0.70, 95% CI = [0.58, 0.82], *p* < 0.001), but did not predict parental delay aversion at T2 (β = -0.06, 95% CI = [-0.18, 0.05], *p* = 0.271). Similarly, parent delay aversion at T1 was predictive of parent delay aversion at T2 (β = 0.71, 95% CI = [0.61, 0.81], *p* < 0.001), but not for child delay aversion at T2 (β = -0.03, 95% CI = [-0.16, 0.09], *p* = 0.612).

## Discussion

The ability to control you own actions and impulses is a fundamental aspect of human development that significantly impacts various domains of functioning through childhood and adolescence. Our study aimed to investigate the relationship between parental and child executive function impairments and delay aversion. The overall aim of the present study was to estimate the association between parents’ self-perceived levels of self-regulatory skills and their assessments of their children’s self-regulatory capacities as well as the longitudinal relations between these abilities.

Our findings demonstrate a significant relationship between the trait factors representing parental and child executive functioning deficits, as well as those representing parental and child delay aversion. Specifically, our models showed substantial shared variance between parental and child assessments of executive function problems and delay aversion. Moreover, results revealed predictive relationships between executive functioning deficits and delay aversion at different measurement time points. Whereas deficits in children’s executive functions and delay aversion at T1 only predicted children’s deficits at T2, and parental delay aversion at T1 only predicted parental delay aversion at T2, parental deficits in executive functions at T1 predicted both parental as well as child deficits in executive functions at T2.

These findings indicate that higher levels of executive function problems reported by parents at T1 correspond to an increased perception of similar problems in their children at T2. This observation is significant as it implies that parents who identify numerous difficulties concerning their own executive functions as well as delay aversion initially are likely to anticipate similar challenges in their children later in development, or alternatively, perceive such difficulties in their children more sensitively. Accordingly, parents’ self-perception of their own skills appears to influence their assessment of their children’s abilities. If parents perceive themselves as well-regulated, they are more likely to rate their children similarly. It seems as if parents draw direct conclusions from themselves to their children.

To our knowledge, this is the first study to focus on the extent to which parental perceptions of their own and their children’s executive function deficits and delay aversion influence the outcomes, rather than focusing on the actual relationship between parents’ and children’s executive function deficits and delay aversion. Moreover, it is also the first investigation that examines this connection generally and longitudinally. Our results are in line with previous findings [35] that propose that parental beliefs, in addition to parental behavior, play a role in shaping child outcomes. More specifically, according to Murphey’s model [35], parental beliefs might influence how parents perceive their children’s behaviors and corresponding outcomes, potentially moderating parental responses accordingly. In addition, our results align with existing literature highlighting the role of parental self-regulatory abilities in child development [60]. We extend the framework of Cuevas and colleagues [60] by revealing intergenerational ties not only within mother-child executive function associations in early childhood, but across parental genders and child age groups. Parents serve as primary models for children’s self-regulatory behaviors, and our findings indicate that parental beliefs about their own self-regulatory skills influence the perceptions of their children’s substantially. The present study added new information by showing that this relationship, at least for executive function deficits, remains stable over time. Our findings go beyond previous research by highlighting the stability of these constructs throughout development. The relationship between parental and child executive functions is robust, implying that even after reassessment several months later, parents’ rating of their children is dependent on their self-ratings, regardless of the severity of initially observed deficits.

As we did not observe such an intergenerational correlation over time for delay aversion, it prompts inquiry into the underlying factors contributing to this discrepancy. One potential explanation for this phenomenon might be that delay aversion was less salient in daily life during the pandemic and therefore less frequently encountered. Furthermore, delay aversion was not recorded as comprehensively within the study as executive function deficits. Since delay aversion is a complex neuropsychological factor that comprises several dimensions [4, 61] it is perhaps more difficult to capture (especially within a survey) than executive functions.

### Strengths and limitations

One of the main strengths of the current study is the large sample size that enhances the generalizability of our findings and provides robust statistical power for detecting relationships between variables. While prior research has predominantly focused on specific age cohorts such as infants [43], children [41], or adolescents [42], our sample encompasses individuals across multiple age groups. This broad inclusion facilitates a more holistic perspective on the topic. Furthermore, assessments were conducted during school closures instead of relying on retrospective reports, providing real-time insights into the impact of distance learning on executive function and delay aversion. In contrast to previous research that has predominantly focused on maternal abilities, our study extends this focus and includes paternal contributions as well. As noted by Ribner and colleagues [43] and Jester and colleagues [42], paternal skills contribute to the association between parental and child self-regulatory skills, too. In the present study, we examine the interrelations across both parental genders rather than isolating analyses to each gender individually, although it is important to note, that significantly more mothers participated in the study.

Our study is the first to examine intergenerational connections longitudinally. Moreover, existing studies have mainly focused on the familial effects on executive functions [41, 43, 60] rather than delay aversion. Here, we also consider delay aversion, as it is equally relevant in the context of self-regulatory abilities [4, 62].

At the same time, several limitations within this investigation should be acknowledged. Firstly, the sole reliance on parental self-report measures concerning child executive functions and delay aversion may introduce bias [18], as parents may overestimate or underestimate their children’s abilities as well as their impact on those abilities. Future research should incorporate multi-informant assessments (e.g., incorporation of teacher reports), direct observations and neuropsychological testing to provide a more comprehensive understanding of parent-child dynamics and to avoid reporter-bias. Direct assessments, such as standardized neuropsychological tests and behavioral observations, provide objective data that can validate and enhance self-report measures. These methods can reduce the subjectivity and potential biases inherent in parental reports. Additionally, the integration of teacher reports can provide a broader context for understanding children’s executive functions and delay aversion. These comprehensive assessments could improve the accuracy and reliability of our findings and contribute to a deeper understanding of the dynamics between parents’ and children’s executive functions and delay aversion. Delay aversion, in particular, is a complex construct that is challenging to accurately quantify [4, 61]. Consequently, future research should aim to develop more comprehensive methods for its measurement.

Secondly, a further limitation is the lack of extensive standardized, validated measurements. Since incorporating numerous scales would have considerably increased the survey length and potentially reduced the response rate, especially among families dealing with mental health issues, only an abbreviated version of all three questionnaires (CHEXI, ADEXI, QDQ) was used. To capture the factors more comprehensively, future investigations should consider incorporating the broader questionnaires and objective tests that capture executive function [7], attentional control [63] and delay aversion [64].

Thirdly, the correlational approach limits our ability to establish causal relationships between parental and child executive functions and delay aversion. Subsequent research should explore the impact of other environmental factors on parent-child interactions and the development of executive functions and delay aversion.

### Implications

Despite these limitations, our study has important implications for both research and practice. By highlighting the significant correlations and longitudinal associations between parental and child executive functions and delay aversion, our findings underscore the importance of considering family dynamics in interventions aimed at promoting executive functions and their ability to withstand delayed reward in children. In view of the fact that parental attributions and expectations influence their children’s treatment progress [44], interventions targeting parental executive functions and delay aversion may indirectly benefit children’s development [29, 60], while interventions directly targeting children may have spillover effects on their parent’s skills [38]. Schneider and colleagues [65], for instance, found that successful treatment of parents’ anxiety disorder is a significant predictor of a better outcome for children’s anxiety sensitivity and agoraphobic cognitions and that even the mere treatment participation (regardless of whether it was successful or not) had a significant positive effect on descendants.

As mentioned earlier, to the best of our knowledge, this study is the first to analyze longitudinal familial relationships based on parents’ self-assessments and their evaluations of their children, rather than on direct comparison of parent and child executive functions and delay aversion. This method enables us to analyze the relationship implied by parents between their own abilities and those of their children, rather than the direct correlation between parent and child skills. The results provide important insights into parental expectations and self-perceptions, contributing to a deeper understanding of the implicit beliefs and assumptions parents have regarding the influence of their abilities on their children’s development. This can serve as an initial motivation to investigate whether and how parental self-assessment correlates with the actual abilities and performance of their children. Future studies should combine both approaches by collecting neuropsychological data from parents and children, as well as parents’ evaluations of their own and their children’s abilities, and vice versa. In light of the study’s findings indicating that parents derive perceptions of their children from their own characteristics, it becomes imperative to consider this phenomenon within therapeutic contexts as well. Our results suggest that early identification and support for children through differentiated diagnostical instruments and the usage of programs specifically designed for the treatment of executive function deficits and delay aversion may help mitigate long-term impacts on academic and socio-emotional outcomes.

Additionally, parents’ self-perception is a key factor in how they assess their children’s condition. Given the connection between parents’ rating of their own and their children’s self-regulatory abilities, it becomes evident that parental skills significantly influence children’s development. Since parents’ ability to adapt their behavior in response to cues and information to meet their children’s current needs is fundamental to effective parenting [66], this underscores the importance of involving parents in interventions aimed at improving children’s self-regulatory skills. By enhancing parents’ cognitive, behavioral, and emotional processes (e.g., planning, emotion regulation, and problem-solving) through targeted parental training, a more supportive environment that promotes positive outcomes for children can be fostered. Thus, incorporating parental training into treatment programs is a critical step in improving children’s development. However, it is important to recognize that parents with poor self-regulatory skills may derive less benefit from conventional parent training compared to those with stronger self-regulatory abilities. Their capacity to implement recommended strategies may be constrained by these deficits. Therefore, it is crucial not only to involve parents in the intervention process but also to tailor the approach to their specific needs. This may include adapting training modules to support parents with executive function deficits and delay aversion more appropriately.

As already addressed in prior studies [66], working closely with significant involved in children’s care or education is crucial for improving self-regulatory skills and should be prioritized more in future practice.

## Conclusion

In conclusion, our study provides evidence of significant correlations and longitudinal associations between parental and child executive functions and delay aversion, emphasizing the role of family dynamics in shaping self-regulatory skills during childhood and adolescence. Despite the constraints of our study, our findings contribute to a deeper understanding of the intergenerational connection of executive functions and underscore the significance of parental inferences regarding their own abilities in relation to those of their children. By these results, a better understanding of the structural covariance and temporal stability of caregivers’ assessments is provided. This has important implications for interventions aimed at promoting positive developmental outcomes in children as well as for therapeutic work. Future research should continue to explore the complex interactions of parental influences, child development, and environmental factors, aiming to develop more effective interventions and support strategies. Specifically, efforts should be made to better target parental involvement in treatment as a way to prevent childhood issues. Therefore, executive functions and delay aversion should be examined from different perspectives by conducting neuropsychological tests of the constructs in addition to detailed questionnaire data. In addition to parents’ assessments of their own and their children’s abilities, children should also assess their own and their parents’ abilities to undertake a multifactorial comparison. Combining the approach of previous studies (measuring children’s and parents’ executive functions and delay aversion) with the approach of this study (measuring parental assessment of parental and child executive functions and delay aversion) and adding children’s assessment of child and parental executive functions and delay aversion could help to determine the extent to which parental assessment predicts or influences children’s actual performance. Moreover, future research should also focus on examining potential differences related to the gender of both children and parents. While our study did not separately analyze mothers and fathers due to the complexity and scope of our models, we acknowledge the importance of this consideration. Therefore, future studies should investigate these gender-specific influences, exploring how maternal and paternal assessments may differ and how these differences impact child development, as well as examining potential variations between male and female children.

## Data Availability

The dataset and corde scripts supporting the conclusions of this article is available in the OSF repository (DOI 10.17605/OSF.IO/RC934) under https://osf.io/rc934/?view_only=5cb3c5e1d5aa4dc0bf25aa78c752dc3c.
